# Clinical manifestation of acute cerebral infarcts in multiple arterial territories

**DOI:** 10.1002/brb3.2296

**Published:** 2021-08-01

**Authors:** Vojtech Novotny, Sander Johan Aarli, Andrej Netland Khanevski, Anna Therese Bjerkreim, Christopher Elnan Kvistad, Annette Fromm, Ulrike Waje‐Andreassen, Halvor Naess, Lars Thomassen, Nicola Logallo

**Affiliations:** ^1^ Department of Neurology Haukeland University Hospital Bergen Norway; ^2^ Department of Clinical Medicine University of Bergen Bergen Norway; ^3^ Centre for Age‐related Medicine Stavanger University Hospital Stavanger Norway; ^4^ Department of Neurosurgery Haukeland University Hospital Bergen Norway

**Keywords:** diffusion‐weighted imaging, multiple stroke, neurological manifestation, symptoms and signs

## Abstract

**Objectives:**

We aimed to assess frequencies and radiological aspects of single‐ and multiterritory clinical manifestation among patients with acute cerebral infarcts in multiple arterial territories (MACI).

**Materials & methods:**

We retrospectively reviewed admission records and diffusion‐weighted magnetic resonance imaging of patients with MACI admitted to our stroke unit between 2006 and 2017. MACI was defined as acute cerebral ischemic lesions in at least two out of three arterial cerebral territories, that is, the left anterior, right anterior and the bilateral posterior territory. Patients with single‐ and multiterritory clinical manifestation were then compared for topographical distribution of the ischemic lesions, the number of ischemic lesions, and The Oxfordshire Community Stroke Project classification.

**Results:**

Out of 311 patients with MACI, 222 (71.4%) presented with single‐territory clinical manifestation. Involvement of the left hemisphere (OR = 0.37, 95% CI 0.16–0.82), less than five ischemic lesions (OR = 0.58, 95% CI 0.35–0.97), and partial anterior circulation infarct clinical stroke syndrome (OR = 0.57, 95% CI 0.34–0.97) were associated with single‐territory clinical manifestation. Involvement of all three territories (OR = 2.58, 95% = 1.48–4.50), more than 10 ischemic lesions (OR = 2.30, 95% CI 1.32–4.01) and total anterior circulation infarct clinical stroke syndrome (OR = 3.31, 95% CI 1.39–7.86) were associated with multiterritory clinical manifestation.

**Conclusion:**

Most patients with MACI present with single‐territory clinical manifestation on admission. Diffusion‐weighted magnetic resonance imaging is therefore necessary for a definite diagnosis.

## Graphical abstract

We aimed to assess frequencies and radiological aspects of single‐ and multi‐territory clinical manifestation among patients with acute cerebral infarcts in multiple arterial territories (MACI). The results show that most patients with MACI present with single‐territory clinical manifestation on admission. Diffusion‐weighted magnetic resonance imaging is therefore necessary for definite diagnosis.

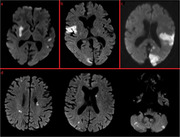

## 1 INTRODUCTION

The clinical manifestation of ischemic stroke may be very diverse depending on the affected area of the brain. In the majority of ischemic stroke patients, brain imaging shows an acute cerebral infarct in a single arterial territory (SACI). With the exception of silent stroke, SACI usually results in a sudden neurological focal dysfunction related to the affected area of the brain.

However, 10−24% of ischemic stroke patients have acute cerebral infarcts in multiple arterial territories (MACI) on diffusion‐weighted magnetic resonance imaging (DWI‐MRI)(Depuydt et al., 2014; Erdur et al., 2019; Novotny et al., 2019). The clinical manifestation of MACI may be unusual due to the simultaneous involvement of several arterial territories, but some patients may present with a single‐territory clinical manifestation even though MACI is found on DWI‐MRI (Depuydt et al., 2014).

The main objective of this study was to assess the frequencies of single‐ and multiterritory clinical manifestation on admission, among patients diagnosed with MACI on DWI‐MRI. We also assessed the relationship between radiological features and clinical manifestation.

## 2 PATIENTS AND METHODS

**FIGURE 1:**
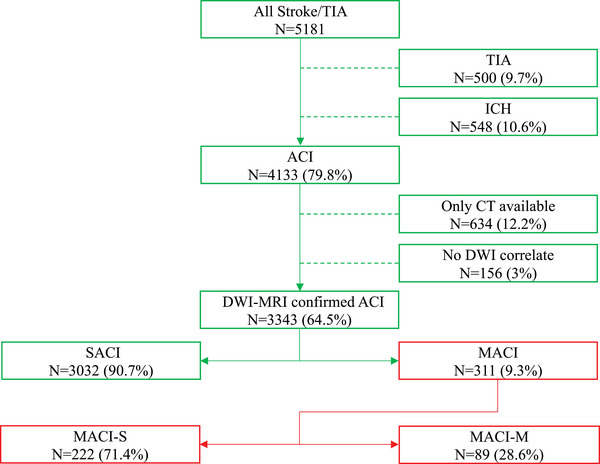
Flowchart of patients included in the study Abbreviations: ACI, acute cerebral infarct; DWI‐MRI, diffusion weighted magnetic resonance imaging; ICH, intracranial hemorrhage; MACI, acute cerebral infarct(s) in a multiple arterial territories; MACI‐M, acute cerebral infarct(s) in multiple arterial territories with multiterritory clinical manifestation; MACI‐S, acute cerebral infarct(s) in multiple arterial territories with single‐territory clinical manifestation; SACI, acute cerebral infarct(s) in a single arterial territory; TIA, transient ischemic attack.

We conducted a retrospective analysis of 311 patients with MACI confirmed on DWI‐MRI. These patients were selected from a cohort of 3343 patients with acute cerebral infarcts admitted to the stroke unit at Haukeland University Hospital between 2006 and 2017. The flowchart of the included patients is shown in Figure 1.

**FIGURE 2:**
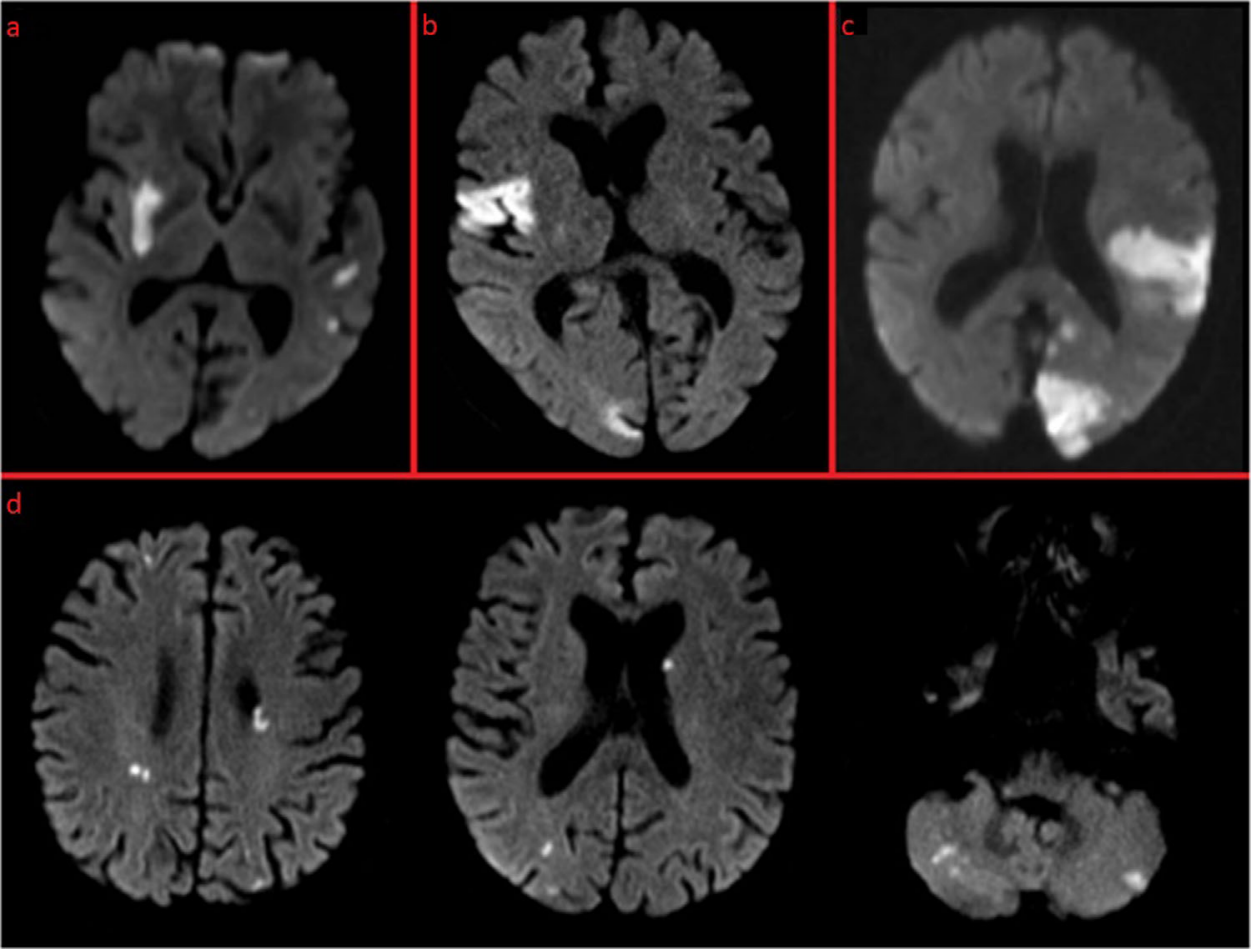
The combinations of affected arterial territories based on the MACI definition. (A) Multiple acute cerebral infarcts distributed in both anterior territories. (b) Multiple acute cerebral infarcts distributed in the right anterior territory and in the posterior territory. (c) Multiple acute cerebral infarcts distributed in the left anterior territory and in the posterior territory. (B) Multiple acute cerebral infarcts distributed in all three territories

MACI was radiologically defined as more than one noncontiguous, acute ischemic lesion in at least two out of three arterial cerebral territories, that is, two anterior territories supplied by the left and by the right carotid artery respectively, and one common posterior territory supplied by the vertebrobasilar arteries. The radiological findings were categorized into four groups based on the combination of the affected territories as left anterior + posterior territory (LAPT), right anterior + posterior territory (RAPT), left + right anterior territory (LRAT) or all three territories (LRAPT) (Tatu et al., 2012) (Figure 2). The number of lesions was categorized into three groups; less than 5, 5–10, and more than 10. The clinical manifestations were categorized into seven groups, four multiterritory originating either from LAPT, RAPT, LRAT, or LRAPT, and three seemingly single‐territory originating either from left anterior territory, right anterior territory or posterior territory alone.

The patients were assigned either to the group presenting with a single‐territory clinical manifestation (MACI‐S) or to the group presenting with multiterritory clinical manifestation (MACI‐M).

The Oxfordshire Community Stroke Project (OCSP) categorization was performed by a stroke neurologist as follows: total anterior circulation infarct (TACI) clinical stroke syndrome, partial anterior circulation infarct (PACI) clinical stroke syndrome, lacunar infarct clinical stroke (LACI) syndrome and posterior circulation infarct (POCI) clinical stroke syndrome (Bamford et al., 1991).

Patients with MACI caused by anatomical variations such as fetal PCA (PCA originating from the carotid artery) and/or azygos ACA (one missing A1‐segment) and/or cross‐flow phenomenon were kept in the analysis.

Two PhD fellows in stroke medicine (VN & SA) independently reviewed admission medical records together with DWI‐MRI scans of all patients with MACI. Clinical manifestation of each patient as described in the medical records on admission was then compared with the topographical distribution of ischemic lesions on DWI‐MRI.

The first 50 patients were reviewed simultaneously by NV and SA using a common framework for clinical stroke manifestations to reach a methodical agreement (Paciaroni et al., 2012; Tatu et al., 2012). The rest of the 261 patients were then assessed by each observer separately. The results were later compared, and in case of disagreement between the two observers, an agreement was reached by a new joint review of those cases, and the final consensus was used for analyses.

The information about the acute clinical manifestation and pre‐existing neurological deficits of each patient were gathered from the initial clinical evaluation performed in the emergency room by the on‐duty neurology resident. The initial clinical evaluation was based essentially on The National Institutes of Health Stroke Scale (NIHSS) (Brott et al., 1989). We also reviewed a more comprehensive clinical evaluation performed by a stroke neurologist the same day or the day after admission. The variable MACI is prespecified in the registry and the population was selected based on the MRI findings performed by on‐duty neuroradiologist and stroke neurologist.

In the majority of patients, the DWI‐MRI was performed by 1.5 Tesla MRI Siemens scanners (Siemens Magnetom Symphony) with a slice thickness of 5 mm and 1 mm gap, field of view 230 × 230 mm, repetition time (TR) 3700 ms and echo time (TE) 89 ms. A minority of patients were examined by 3.0 Tesla MRI Siemens scanner (Siemens Magnetom Skyra and Prisma) with slice thickness 4 mm and 1.2 mm gap, field of view 220 × 220 mm, repetition time (TR) 3400 ms and echo time (TE) 54–89 ms.

Only DWI‐MRI lesions appearing as hyperintense signals accompanied by hypointense signals on apparent diffusion coefficient magnetic resonance imaging (ADC‐MRI) were considered as acute. Patients with slightly hyperintense DWI‐MRI signals or those DWI‐MRI signals without corresponding ADC‐MRI changes were considered as subacute lesions or artefacts.

The supplementary data that support the findings of this study are available from the corresponding author on reasonable request. The Bergen NORSTROKE registry has been approved by local ethics committee REK West and written consent has been obtained from study participants or their legal representatives.

### 2.1 Statistical analysis

For the univariate analyses, we used Mann–Whitney *U* test, *t*‐test or chi‐squared test depending on the skewness of the data and type of the analyzed variables. We performed multivariable logistic regression analyses adjusted for relevant clinical confounders, including NIHSS on admission, sex and age to obtain odds ratios together with 95% confidence intervals for distribution of the lesions, number of the lesions and OCSP categories. MACI‐M/MACI‐S was used as the dichotomous outcome (dependent) variable. The significance level was set to ≤.05. We used STATA 15.0 software (STATA Corp. TX) for all analyses.

## 3 RESULTS

In the cohort of 311 patients with MACI confirmed on DWI‐MRI, 222 (71.4%) patients had single‐territory clinical manifestation (MACI‐S). These patients had lower median NIHSS on admission compared to the patients with multiterritory clinical manifestation (MACI‐M) [3 (IQR = 1–8) versus 5 (IQR = 2–11)]. The demographics and other baseline characteristics were otherwise balanced between both groups, including the time from stroke onset to MRI examination (Table 1).

**TABLE 1:** Baseline characteristics, radiological findings, clinical‐etiological classification

	MACI‐S*N* = 222	MACI‐M*N* = 89	*p*‐Value
Demographics			
Age, mean (SD), y	72.8 (12.2)	72.1 (14.8)	0.651
Females, *N* (%)	104 (46.9)	44 (49.4)	0.679
Patient characteristics			
NIHSS score, median (IQR)	3 (1–8)	5 (2–11)	0.036
Prestroke mRS, median (IQR)	0 (0–1)	0 (0–2)	0.679
SBP, mean (SD), mmHg	158.4 (28.2)	157.7 (28.5)	0.839
DBP, mean (SD), mmHg	85.4 (16.2)	86.9 (15.8)	0.459
Temperature, mean (SD),°C	36.6 (1.0)	36.8 (0.8)	0.069
O_2_ Saturation, mean (SD), %	96.7 (3.1)	96.5 (3.4)	0.515
Blood glucose, mean (SD), mmol/L	6.9 (2.4)	7.2 (3.1)	0.415
Time from stoke onset to MRI, median (IQR), days	1 (1–3)	1 (1–3)	0.996
TOAST classification			
Cardiogenic embolism, *N* (%)	120 (54.1)	44 (49.4)	0.461
Large‐vessel disease, *N* (%)	17 (7.7)	8 (9.0)	0.696
Small‐vessel disease, *N* (%)	5 (2.3)	4 (4.5)	0.286
Other determined cause, *N* (%)	5 (2.3)	4 (4.5)	0.286
Undetermined cause, *N* (%)	75 (33.8)	29 (32.6)	0.839
The OCSP classification			
TACI, *N* (%)	30 (13.5)	26 (29.2)	0.001
PACI, *N* (%)	105 (47.3)	29 (32.6)	0.018
LACI, *N* (%)	41 (18.5)	13 (14.6)	0.416
POCI, *N* (%)	46 (20.7)	21 (23.6)	0.577
Topography of lesions			
LAPT, *N* (%)	46 (20.7)	8 (9.0)	0.014
RAPT, *N* (%)	40 (18.0)	11 (12.4)	0.223
LRAT, *N* (%)	96 (43.2)	37 (41.6)	0.788
LRAPT, *N* (%)	40 (18.0)	33 (37.1)	<0.001
Number of lesions			
< 5	121 (54.5)	36 (40.5)	0.025
5–10	58 (26.1)	21 (23.6)	0.643
> 10	43 (19.4)	32 (36.0)	0.002

Abbreviations: LACI, lacunar infarct clinical stroke syndrome; LAPT, left anterior + posterior territory; LRAPT, all 3 territories; LRAT, left + right anterior territory; MACI‐M, acute cerebral infarcts in multiple arterial territories presenting with multiterritory clinical manifestation; MACI‐S, acute cerebral infarcts in multiple arterial territories presenting with single‐territory clinical manifestation; MRI, magnetic resonance imaging; NIHSS, National Institutes of Health Stroke Scale; OCSP, The Oxfordshire Community Stroke Project; PACI, partial anterior circulation infarct clinical stroke syndrome; POCI, posterior circulation infarct clinical stroke syndrome; RAPT, right anterior + posterior territory; TACI, total anterior circulation infarct clinical stroke syndrome; TOAST, Trial of ORG 10172 in Acute Stroke Treatment.

MACI distributed in LAPT were associated with single‐territory clinical manifestation (OR = 0.37, 95% CI 0.16–0.82) and MACI distributed in LRAPT were associated with multiterritory clinical manifestation (OR = 2.58, 95% CI = 1.49–4.50) (Table 2).

**TABLE 2:** Multivariable regression analyses with MACI‐M / MACI‐S as an outcome variable

	OR^†^	95% CI	*p*‐Value
The OCSP classification			
TACI, *N* (%)	3.31	1.39–7.86	0.007
PACI, *N* (%)	0.57	0.34–0.97	0.039
LACI, *N* (%)	0.82	0.41–1.63	0.561
POCI, *N* (%)	1.39	0.75–2.57	0.294
Topography of lesions			
LAPT, *N* (%)	0.37	0.16–0.82	0.015
RAPT, *N* (%)	0.67	0.32–1.37	0.265
LRAT, *N* (%)	0.95	0.58–1.58	0.849
LRAPT, *N* (%)	2.58	1.49–4.50	0.001
Number of lesions			
0–5	0.58	0.35–0.97	0.040
5–10	0.84	0.47–1.50	0.549
> 10	2.30	1.32–4.01	0.003

Abbreviations: CI, confidence interval; LACI, lacunar infarct clinical stroke syndrome; LAPT, left anterior + posterior territory; LRAPT, all 3 territories; LRAT, left + right anterior territory; OCSP, The Oxfordshire Community Stroke Project; OR, odds ratio; PACI, partial anterior circulation infarct clinical stroke syndrome; POCI, posterior circulation infarct clinical stroke syndrome; RAPT, right anterior + posterior territory; TACI, total anterior circulation infarct clinical stroke syndrome .

^†^ Dependent (outcome) variable: MACI‐M/MACI‐S; Independent (adjustment) variables: NIHSS on admission, sex and age.

Patients with less than five ischemic lesions more often presented with single‐territory clinical manifestation (OR = 0.58, 95% CI 0.35–0.97) while patients who had more than 10 ischemic lesions more often presented with multiterritory clinical manifestation (OR = 2.3, 95% CI 1.32–4.01) (Table 2).

The patients with MACI‐S were more likely to be assigned to the PACI clinical stroke syndrome (OR = 0.57, 95% CI 0.34–0.97) whereas the patients with MACI‐M were more likely to be assigned to the TACI clinical stroke syndrome (OR = 3.31, 95% CI 1.39–7.86) (Table 2). The seven possible combinations of clinical manifestations in relation to the distribution of the ischemic lesions are summarized in Table 3.

**TABLE 3:** Topography of MACI in relation to clinical manifestation

Origin of clinical manifestation		Topography of MACI				Total
		LAPT *N* = 54 (%)	RAPT *N* = 51 (%)	LRAT *N* = 133 (%)	LRAPT *N* = 73 (%)	*N* = 311
MACI‐S	LT	28 (51.8)	0 (0)	58 (43.6)	11 (15.1)	97
	RT	1 (1.9)	26 (51)	38 (28.6)	17 (23.3)	82
	PT	17 (31.5)	14 (27.5)	0 (0)	12 (16.4)	43
MACI‐M	LAPT	8 (14.8)	0 (0)	0 (0)	6 (8.2)	14
	RAPT	0 (0)	11 (21.5)	0 (0)	13 (17.8)	24
	LRAT	0 (0)	0 (0)	37 (27.8)	8 (11)	45
	LRAPT	0 (0)	0 (0)	0 (0)	6 (8.2)	6

Abbreviations: LAPT, left anterior + posterior territory; LRAPT, all 3 territories; LRAT, left + right anterior territory; LT, left anterior territory; MACI, acute cerebral infarcts in multiple arterial territories; MACI‐M, acute cerebral infarcts in multiple arterial territories presenting with multiterritory clinical manifestation; MACI‐S, acute cerebral infarcts in multiple arterial territories presenting with single‐territory clinical manifestation; PT, posterior territory; RAPT, right anterior + posterior territory; RT, right anterior territory.

## 4 DISCUSSION

In this cohort study of patients with MACI, we compared the clinical manifestation as described in the admission records with the topographic distribution of the ischemic lesions as detected by DWI‐MRI.

Over 70% of patients with MACI presented with clinical manifestation corresponding to only one of the affected arterial territories. There may be several explanations for this. It is probable that some of the acute lesions were clinically silent. The frequency of silent stroke in elderly population may be around 20% (Vermeer et al., 2007), but the frequency of acute silent stroke is unknown. The frequency of both silent stroke and MACI increases with higher age (Novotny, Thomassen et al., 2017; Vermeer et al., 2003), which may contribute to our finding. Many silent lesions are truly asymptomatic, but some of them may cause neurological deficit in form of a subtle cognitive deterioration (Lei et al., 2019), not noticed by the patient, the physician, or the patient's family. In large artery atherosclerosis‐associated MACI, the ischemic events may be separated by hours or days because of successive embolization (Novotny, Khanevsky et al., 2017), which may influence the final clinical assessment or the time of the MRI examination. However, we found no difference in the time from stroke onset to MRI examination either between patients with SACI (*N* = 3032) and MACI (*N* = 311) or between patients with MACI‐S (*N* = 222) and MACI‐M (*N* = 89) (Novotny et al., 2020) (Table 1).

Another explanation for our finding may be the insufficiency of NIHSS regarding lesions in the posterior circulation compared to the anterior circulation. Thus, symptoms originating from the posterior circulation may be underdiagnosed (Sato et al., 2008). Also, severe neurological deficit from one hemisphere may shadow subtle symptoms originating from another arterial territory. NIHSS favors the left, mostly dominant hemisphere over the right, mostly nondominant hemisphere (Fink et al., 2002; Portegies et al., 2015; Woo et al., 1999). Cognitive symptoms originating from lesions in the right anterior territory may be more difficult to recognize than the deficits in language and dominant extremity function originating in most cases from the left anterior territory (Foerch et al., 2005; Puig‐Pijoan et al., 2018). This reflects the overweight of left anterior territory symptoms alone among patients with lesions involving both left and right anterior territories (43.6% vs. 28.6%) (Table 3).

MACI distributed in all three territories were more likely to present with multiple‐territory clinical manifestation (Table 2), conceivably because there are three possible combinations of multiterritory clinical manifestations, namely from LAPT, RAPT or all three territories (LRAPT). The frequencies of MACI distributed in LAPT and RAPT was almost equal in our study (17.4% vs. 16.4%). The involvement of the left arterial territory was, however, associated with single‐territory clinical manifestation, which again emphasizes the dominance of the left‐hemisphere symptoms (Table 2).

As one would expect, increasing number of lesions may favor the multiterritory clinical manifestation and our finding is in line with this hypothesis (Table 3).

The cerebral infarcts in MACI presenting with multiterritory clinical manifestation (MACI‐M) were more often categorized as TACI clinical stroke syndrome according to the OCSP classification. This fact and higher number of lesions also reflect higher NIHSS on admission thus higher occurrence of a more severe stroke in this group (Tables 1 & 2). Thus, our findings suggest that more severe stroke may increase the odds for recognition of patients with MACI‐M.

This study shows that DWI‐MRI is essential for the final diagnosis of MACI whereas the clinical manifestation is not reliable in most cases. MACI is an important diagnosis, since cardiogenic embolism represents the primary etiology in this group (Baird et al., 2000; Novotny, Thomassen, et al., 2017). Recognition of cardiogenic embolism is crucial for early secondary prevention with anticoagulants. Where DWI‐MRI is not readily available, highly qualified clinical neurological examination with awareness of possible MACI is needed in acute stroke. However, there is so far no study supporting that another approach in neurological examination in acute settings may reveal such patients.

This study has some limitations. First, the study was clinically retrospective, based on the medical records performed by different physicians, which could bring inaccuracy in regards to the clinical manifestation assessment. On the other hand, the study represents real life standard neurological assessments of stroke patients on admission. In over 70% of our patients, concomitant symptoms originating from other arterial territories may have been unrecognized or not present. However, our results are in line with the results of Depuydt and his colleagues who reported almost identical rate of MACI‐S and MACI‐M (70% vs. 30%) (Depuydt et al., 2014). Second, the assessment of clinical manifestation in acute setting is mostly based on the NIHSS, which has limited ability for recognition of neuropsychological symptoms, symptoms from the posterior circulation and from the right hemisphere (Fink, 2005). Third, the comparison of the MRI‐DWI findings and clinical manifestation was not blinded with respect to each other, which might have caused a selection bias. However, similar neurological deficits may be caused by lesions located in different brain areas or even cerebral territories. We therefore decided to avoid such misclassifications by nonblinded review performed by two independent assessors. Both the size and possibly even the presence of the lesions may be influenced by the initial treatment but also by the time from the stroke onset to MRI examination. Acute MRI in all included patients would therefore be preferable in the setting of our study. Finally, we have no data on ischemic lesion volumes which could have influenced clinical manifestation, large ischemic lesions yielding neurological symptoms whereas microlesions remained clinically undetected.

## 5 CONCLUSION

The majority of patients with MACI presented with a single‐territory clinical manifestation on admission. Single‐territory clinical manifestation is associated with involvement of the left anterior territory, less than five lesions in total and PACI clinical stroke syndrome as defined in OCSP classification. Multiple‐territory clinical manifestation is associated with involvement of all three territories, more than 10 lesions in total and TACI clinical stroke syndrome as defined in OCSP classification. The clinical inability to define MACI patients shows that the diagnosis needs DWI‐MRI verification in most cases.

## FUNDING INFORMATION

This work was supported by the University of Bergen, which had no influence on the study design, data collection and presentation or the conclusions made. Projekt number: REK 2012/1483.

## CONFLICT OF INTEREST

The authors declare no conflict of interest.

## Data Availability

The data that support the findings of this study are available from the corresponding author upon reasonable request.
